# Invest in health and uphold rights to “build back better” after COVID-19

**DOI:** 10.1080/26410397.2020.1781583

**Published:** 2020-07-10

**Authors:** Helen Clark, Anna Gruending

**Affiliations:** a Board Chair, Partnership for Maternal, Newborn & Child Health, World Health Organization, Geneva, Switzerland; Former Prime Minister, New Zealand (1999–2008); b Consultant, Partnership for Maternal, Newborn & Child Health, World Health Organization, WHO, Geneva, Switzerland

**Keywords:** universal health coverage, global health security, sexual and reproductive health and rights, COVID-19, political leadership

## Abstract

The COVID-19 pandemic is not just a health crisis – it is a full-blown economic and social crisis that is impacting the lives and livelihoods of billions of people. This commentary examines the mutually dependent relationship between health security and universal health coverage (UHC), and how the longstanding underinvestment in both renders us all vulnerable. It also discusses the vulnerability of services for sexual and reproductive health and rights (SRHR) in times of crisis, which is compounded when these services are not included and well integrated into national UHC packages. It concludes with a call for stronger political leadership for UHC and SRHR as the global community strives to “build back better” after COVID-19.

## Résumé

La pandémie de COVID-19 n’est pas seulement une crise sanitaire, c’est une véritable crise économique et sociale qui a des conséquences sur la vie et les moyens d’existence de milliards de personnes. Ce commentaire examine la relation mutuellement dépendante entre sécurité sanitaire et couverture santé universelle (CSU) et de quelle manière le sous-investissement chronique dans ces deux domaines nous rend tous vulnérables. Il aborde aussi la vulnérabilité des services de santé et droits sexuels et reproductifs en temps de crise, une vulnérabilité qui est aggravée quand ces services ne sont pas inclus et bien intégrés dans les paniers nationaux de la CSU. Il se termine par un appel pour un leadership politique plus énergique autour de la CSU ainsi que de la santé et des droits sexuels et reproductifs, tandis que la communauté internationale s’efforce de « se reconstruire en mieux » après le COVID-19.

## Resumen

La pandemia de COVID-19 no es solo una crisis de salud, sino también una crisis económica y social generalizada, que está impactando la vida y el sustento de miles de millones de personas. Este comentario examina la relación mutuamente dependiente entre la seguridad de salud y la cobertura universal de salud (CUS), y cómo la subinversión de larga data en ambas hace vulnerables a todas las personas. Además, discute la vulnerabilidad de los servicios de salud y derechos sexuales y reproductivos (SDSR) en tiempos de crisis, que es agravada cuando estos servicios no son incluidos y bien integrados en los paquetes nacionales de CUS. Concluye con un llamado a mejor liderazgo político para la CUS y SDSR, a medida que la comunidad mundial lucha por 'reconstruir mejor' después de COVID-19.

The COVID-19 pandemic is not just a health crisis – it is a full-blown economic and social crisis that is impacting the lives and livelihoods of billions of people. Its spread and our collective response have laid bare stark realities about the dangers of weak health systems and a lack of universal health coverage (UHC), and about the devastating consequences if human rights, including sexual and reproductive health and rights, are not upheld.

First, COVID-19 has exposed the mutually dependent relationship between health security and UHC, and how the longstanding underinvestment in both renders us all vulnerable. Global health security refers to activities, both proactive and reactive, to minimise the danger and impact of public health events on people’s health across regions and international boundaries. COVID-19 is just the latest example of how an outbreak anywhere can carry enormous risk everywhere. Without proper surveillance and preparedness, outbreaks place an enormous burden on health services. Without equitable and affordable access to health services, people may avoid testing and treatment, heightening the risks of further transmission. Weak health systems and a lack of equitable access to affordable health services and information undermine states’ ability to uphold the right to the highest attainable standard of health. Even in countries with established public health surveillance systems, the lack of a gender and human rights lens can lead to overlooking the differentiated impacts of outbreaks, which often run like fractures along the lines of pre-existing inequities.

UHC – access to needed health services without the risk of catastrophic impoverishment – sadly remains a distant dream for millions. The World Health Organization (WHO) estimates that at least half of the world’s population do not have full coverage of essential health services, and roughly 100 million people are pushed into extreme poverty because of health care costs.^1^

Even in countries with UHC, there are often co-payments for services which disproportionately impact on the poor, leading to a delay in seeking care or to outright inability to seek it. The OECD recently reported that in 23 of their member countries, 17% of people forego care because costs are too high.^2^ There are also significant gendered impacts, with women providing the lion’s share of unpaid care that isn’t “covered” by the health system.^3^ When it comes to infectious diseases, the delay or inability to seek testing and treatment puts an extra burden on women and can be deadly for individuals, and indeed for all of society.

Across the globe, we have seen COVID-19 exacerbate existing health disparities. In the United Kingdom, COVID-19 hospital deaths among Britons of black African background are 3.7 times higher than those of white Britons, and British Pakistani deaths are 2.9 higher, after accounting for age, sex and geography.^4^ In Chicago, African Americans account for over 50% of COVID-19 cases and almost 70% of deaths, whereas they make up just 30% of the city’s population.^5^ Some of the disparities exposed may be attributable to poorer health status and/or to over-representation in front-line jobs, reflecting structural disadvantages. Limited access, physical and/or financial, to health services and information, however, surely plays a part.

We have also seen discrimination rear its ugly head during the pandemic, including in health care settings. We know from past pandemics that discrimination contravenes human rights, is detrimental to the health of individuals and communities, and hinders the overall public health response. This has been extensively documented in the HIV/AIDS pandemic, as well as the 2014–2016 Ebola epidemic in West Africa.

Migrants, refugees and internally displaced persons (IDPs) are particularly vulnerable to stigma and discrimination, and many are living in overcrowded settings with inadequate access to sanitation facilities, making social distancing and regular handwashing particularly challenging, if not impossible. Many have limited – if any – access to health care, and may not seek care for fear of being arrested or deported.^6^ Homophobic and transphobic rhetoric has been on the rise in certain countries, and LGBTI persons have been made into scapegoats for spreading the virus. LGBTI people also regularly face stigma and discrimination when accessing health care, which dissuades them from seeking out these services, carrying extra risks during the pandemic.^7^

The current strain on health systems and limited mobility due to lockdowns are also posing specific challenges for people living with HIV/AIDS, compounding discrimination and limiting access to life-saving anti-retroviral medication. Many other populations also face barriers due to stigma and discrimination, including but not limited to Indigenous peoples, persons with disabilities, and racial, ethnic and religious minorities.^6^ In short, discrimination in health care settings disproportionately harms populations who are already marginalised, entrenching inequities.

Governments leading their respective COVID-19 responses must learn from past pandemics. Large-scale investment in scaling up testing for HIV has been transformative for the global HIV/AIDS response, empowering people to take action to protect their own health, and providing states with a critical tool to combat the pandemic. COVID-19 has demonstrated the importance of universal access to free or affordable screening, testing and care, and how lack of access can exacerbate the crisis and hamper the health system response. It has also confirmed – yet again – that non-discrimination must be a central pillar of the public health response, with authorities working in partnership with affected communities.

## Sexual and reproductive health and rights in peril

A second unfortunate truth laid bare by COVID-19 is the vulnerability of services for sexual and reproductive health and rights (SRHR) in times of crisis, disproportionately and unjustly impacting women and girls. Sexual and reproductive health and rights are an integral part of the right to the highest attainable standard of health, and indivisible from other human rights which countries are obliged to uphold under international law. While SRHR services have historically been the most difficult to establish and institutionalise, in times of crisis they seem to be the first to be axed or the last to be protected. When these services are not included in UHC national packages – or sometimes included but not financed – their availability during crises becomes even more precarious.

The continuity of abortion services has been uncertain in various settings, to the detriment of women’s autonomy, health and rights. In India, it was three weeks into the lockdown that the national government declared abortion to be an “essential service.” Rights groups, however, fear that a lack of transportation and mobility still severely limits women’s access, particularly for the poor.^8^ In the United States, multiple states have taken steps, with varying degrees of success, to close abortion clinics or dramatically curtail access during the COVID-19 pandemic. In Poland, the ruling party has been criticised for taking steps during the pandemic to tighten some of the most restrictive abortion laws in Europe further, while activists’ ability to protest is severely limited.

Gender-based violence, one of the most ubiquitous violations of human rights, has emerged as a “shadow pandemic,” with alarming reports from helplines, service organisations and authorities across the globe. Lockdowns increase time spent with abusers in situations of high stress, limit survivors’ access to services and support networks, and shrink their opportunities to leave.^9^ LGBTI persons are at heightened risk of violence, with many LGBTI youth confined at home with unsupportive or even hostile family members.^7^ While some countries have quickly recognised the additional risks posed by the pandemic and acted to address them, most policy measures have been reactive, perhaps reflecting the absence of a gender lens in emergency preparedness plans. Elsewhere, the silence is deafening, including in countries where intimate partner violence isn’t a crime, leaving women and children confined with their abusers without support or legal recourse.

An absence of government action has also left maternal and newborn health (MNH) services vulnerable to ad hoc restrictions, such as forced caesarean sections and mandatory separation of newborns from COVID-19 positive mothers, flying in the face of evidence and global recommendations.^10^ The diversion of resources away from MNH services also places them in jeopardy, and may lead to diminished care and decreased demand, as happened during the 2014–2016 Ebola epidemic in West Africa. In Sierra Leone, antenatal care coverage during the epidemic decreased by 22%. Reduced utilisation of maternal health services is estimated to have resulted in at least 3,600 additional maternal, neonatal and stillbirth deaths in 2014–2015. To put this into perspective, this is equivalent to total direct Ebola deaths in Sierra Leone over the course of the entire epidemic.^11^

Preliminary models predict that the COVID-19 pandemic is likely to result in significant increases in maternal mortality due to reduced service availability. The Guttmacher Institute has estimated that even a modest 10% decline in access to SRHR services in low- and middle-income countries (LMIC) could have disastrous consequences, including an additional 15 million unintended pregnancies, an additional three million unsafe abortions, and an additional 28,000 maternal deaths, 1000 of these due to unsafe abortion.^12^ UNFPA has estimated that for every three months that lockdowns continue, an additional 15 million cases of gender-based violence can be expected in LMIC.^13^

## Building back better after COVID-19

The COVID-19 pandemic has exposed inequities in our societies, as well as the dispensability of human rights and of women’s lives. As argued in this piece, inequities include, but are not limited to, the millions who are threatened by the virus without access to affordable and quality health services, and those who face persistent discrimination which is compounded during the pandemic, including women and girls who suffer unduly because of decreased access to essential SRHR services and information.

Yet times of crisis also provide a chance to address the imbalances and to build stronger and more resilient health systems and partnerships. To “build back better” from COVID-19, we need to put health on the top of global and national agendas, to prioritise UHC with an emphasis on reducing inequities and confronting discrimination, and to enshrine SRHR at the centre of these efforts. This will require bold leadership and global co-operation on an unprecedented scale.

The COVID-19 pandemic has demonstrated the realm of the possible when political leaders grasp the urgency of a situation and take decisive action. It is also a reminder of the importance of government (for those who doubted it!), and the responsibilities that rest with governments in upholding the right to health. For the good of their people and their economies, we need strong political leadership to accelerate the expansion of UHC, in line with commitments made at last year’s UN High-Level Meeting on UHC and with a focus on rights. This, of course, includes meeting the needs of women, adolescents and girls by including a comprehensive SRHR package of services, which is both inexpensive and cost-effective.

Throughout this crisis, many of the world’s female leaders have been particularly inspiring, demonstrating calm, clear, and competent leadership. Key to that successful leadership is openness, transparency, and a commitment to accountability and to key human rights principles. Let us not forget these attributes as we look to build back from this crisis, nor the importance of supporting more women to run for office and take on the top roles.

Leadership isn’t only required at the national level. Global co-operation, partnership and solidarity are what will help us overcome the current crisis, and must also be leveraged to meet other global challenges – including the climate crisis, ensuring health for all, and tackling gender inequality.

Though global co-operation has been notably absent from some of the bodies from which we would expect it, there are glimmers of hope. In late April, WHO and numerous multilateral and private partners launched the “Access to COVID-19 Tools” (ACT) Accelerator, a global collaboration for the development and production of and equitable access to new COVID-19 diagnostics, therapeutics and vaccines. In response, at a pledging summit convened by the European Union, an initial €7.5 billion was promised by countries and other partners. The financial commitment is significant, indicating a spirit of global solidarity, as well as a commitment to equity, ensuring that all people – of every age, gender, and socio-economic status – can benefit from the fruits of this partnership. Importantly, this initiative will also support health system strengthening.

Countries are also mobilising around the UHC and SRHR agenda. In early May, 59 countries issued a joint statement urging the protection of sexual and reproductive health and rights and promoting gender-responsiveness in the COVID-19 crisis. These countries recognise the central role of UHC, and call on all governments to ensure full and unimpeded access to all SRHR services for women and girls.^14^

The returns on investing in UHC, including SRHR, aren’t just a healthier, more productive population whose right to health is upheld, but also better resilience in the face of global health threats. In just seven years – from 2011 to 2018 – the World Health Organization confronted 1483 epidemics,^15^ and so we would be foolhardy to believe that our health systems won’t soon be tested again. Let us all work together to ensure that the legacy of this crisis is one of co-operation, solidarity, and health and human rights for all.
